# Antileishmanial activity of *Urtica dioica* extract against zoonotic cutaneous leishmaniasis

**DOI:** 10.1371/journal.pntd.0007843

**Published:** 2020-01-13

**Authors:** Alireza Badirzadeh, Maryam Heidari-Kharaji, Vahid Fallah-Omrani, Hossein Dabiri, Atefeh Araghi, Alireza Salimi Chirani

**Affiliations:** 1 Department of Parasitology and Mycology, School of Medicine, Iran University of Medical Sciences, Tehran, Iran; 2 Skin and Stem Cell Research Center, Tehran University of Medical Sciences, Tehran, Iran; 3 Cellular and Molecular Biology Research Center, Shahid Beheshti University of Medical Sciences, Tehran, Iran; 4 Department of Medical Microbiology, School of Medicine, Shahid Beheshti University of Medical Sciences, Tehran, Iran; 5 Department of Clinical Sciences, Faculty of Veterinary Medicine, Amol University of Special Modern Technologies, Amol, Iran; Instituto de Ciências Biológicas, Universidade Federal de Minas Gerais, BRAZIL

## Abstract

**Background:**

Neglected parasitic diseases (NTDs) like cutaneous leishmaniasis (CL) have caused high mortality and morbidity rate in developing countries. This disease is considered as one of the six major tropical diseases, and has a great importance in HIV infected individuals as an opportunistic infection in those areas that both infections are endemic. This study evaluated the therapeutic effects of the *Urtica dioica* L (*U*. *dioica*) aqueous extract as an anti-leishmanial herbal drug *in-vitro* and *in-vivo*, and in addition to that, evaluated two vital immune system cytokines including gamma interferon (IFN-γ) and interleukin-4 (IL-4) plus nitric oxide (NO) and arginase activity against *Leishmania major* (*L*. *major*) infected mice.

**Methodology/Principal findings:**

*In-vitro* anti-leishmanial activity of *U*. *dioica* aqueous extract was determined using MTT method and also Parasite Rescue Transformation Assay. Also, the footpad lesion size and parasite load in BALB/c mice infected with *L*. *major* were quantified for *in-vivo* assessment. Furthermore, for evaluating the immune responses, the levels of IFN-γ, IL-4, NO and arginase were measured in the BALB/c mice. These results indicated that *U*. *dioica* extract significantly reduced the *L*. *major* promastigotes viability. According to the *in-vitro* cytotoxicity assay of the extract on *Leishmania* parasites (CC50) and infected macrophages (EC50), the extract had no toxicity to the macrophages, however it efficiently killed the *L*. *major* amastigotes. In addition, the lesion size, parasite load, IL-4, and ARG were decreased in the treated infected mice, however IFN-γ and NO were significantly increased.

**Conclusions/Significance:**

This study established satisfactory results in *Leishmania* parasite clearing both *in-vivo* and *in-vitro*. Therefore, *U*. *dioica* extract can be considered as an effective and harmless herbal compound for killing the parasite without toxicity to the host macrophages. Furthermore, it also can treat the CL by switching the mouse immune response towards a cell-mediated response (Th1); hence, it may be identified as a perfect therapeutic herbal drug for CL treatment.

## Author summary

Nowadays, neglected tropical diseases (NTDs) like leishmaniasis have increased the mortality and morbidity rate in developing countries, especially in tropical regions, and is considered as responsible for more than 20 million deaths per year. Although, cutaneous leishmaniasis (CL) is rapidly spreading in all over the world, but this disease controlling has remained insufficient because of low-cost treatment and the lack of an effective vaccine. Therefore, new treatment approaches for leishmaniasis are immediately required. Up to now, application of natural herbal products has been considered as one of the major effective ways in treating the protozoan parasitic infections like CL. In this study, we evaluated the therapeutic effects of the *Urtica dioica* L (*U*. *dioica*) aqueous extract as an anti-leishmanial herbal drug *in-vitro* and *in-vivo*, and also quantified the immune cytokines IFN-γ, IL-4 against CL caused by *Leishmania major* (*L*. *major*). The *in-vitro* activity of *U*. *dioica* aqueous extract on *L*. *major* was determined using MTT method and Parasite Rescue Transformation Assay. Moreover, the footpad lesion size was assessed and parasite load in the mice infected with *L*. *major* was quantified *for in-vivo*. In order to evaluate the immune responses, IFN-γ, IL-4, NO and arginase were measured in the *Leishmania* infected mice. Accordingly, *U*. *dioica* is one of the effective herbal plant in *L*. *major* parasite clearing without any harmful toxicity to the murine cells, and can strongly cure the zoonotic CL.

## Introduction

Due to the reason that neglected tropical diseases (NTDs) like leishmaniasis have resulted in high mortality and morbidity in developing countries, several species of the protozoan parasites belonging to the genus *Leishmania* are identified as the causative agent that are responsible for leishmaniasis [1, 2]. This disease is one of the main endemic parasitic infections worldwide, which is found in around 98 countries, especially in developing countries with about 1.7 billion people at risk of contracting the infection [3, 4]. Also, leishmaniasis is considered as one of the six major NTD by the World Health Organization (WHO), due to its remarkable effect on global public health, and has great importance in HIV infected individuals as an opportunistic infection in those areas that both infections are endemic [5, 6]. This devastating parasitic infection could be clinically categorized into three significant forms: cutaneous leishmaniasis (CL), visceral leishmaniasis (VL) and muco-cutaneous leishmaniasis (MCL) [7]. Globally, CL is the most widespread form of this disease with approximately one million new cases that are diagnosed annually, and also with about 90% of the cases found in developing countries including Iran, Afghanistan, Algeria, Brazil, Peru, Syria and Saudi Arabia [8, 9].

Despite of the CL global spread, the disease controlling has remained unsatisfactory due to the lack of an effective vaccine and low-cost treatment [10, 11]. In accordance with that, current anti-leishmanial drugs have several disadvantages like toxicity, costliness and drug-resistance [12]. Pentavalent antimonials that are the first-line drugs in the CL treatment could result in severe toxic side effects including cardiotoxicity, pancreatitis, hepatotoxicity and nephrotoxicity [13]. On the other hand, second-line drugs like pentamidine and miltefosine may cause diabetes at the time that they are applied with high doses [14, 15]. Furthermore, resistance that has recently developed against certain antileishmanial drugs lead to treatment failures [16, 17]. Therefore, new treatment approaches are immediately required [18, 19].

Up to now, application of natural herbal products has been one of the major effective ways in the protozoan parasitic infections therapy like CL. Several studies have proposed that natural herbal antiparasitic plants, especially in the case of CL, do not have a strong probability of creating adverse effects. They are economical, with easy access, sustainable, and have considerable immunomodulatory efficacies in comparison with popular antileishmanial drugs. These herbal plants can be utilized as excellent cost-effective options for treatment of some diseases like cancers and parasitic disease, where the human immune responses are vital for the disease development [20]. Approximately 80% of the global population use phytomedicine for infectious and non-infectious diseases treating; therefore, herbal medicine has received noticeable scientific interest, specifically for validating their medicinal usages. Regarding, WHO recently updated the Traditional Medicine Strategy for 2014–2023 to prioritize traditional and complementary medicine products [21, 22].

In folk medicine, one of the less investigated medicinal herbs for its antiparasitic features is *Urtica dioica L*. *(U*. *dioica)*, which is known as stinging nettle or *Urticaceae* and is existing in distinct parts of the world like India, United States, Malaysia, and Iran. This herbal plant has various active compounds including mucilage, tannins, formic acid, wax, tosterin, calcium, iron, potassium nitrate, and more importantly glucoside compounds with a tegumental irritating nature [23, 24]. *U*. *dioica* has different effects including anticancer, anti-inflammation, antirheumatic, cardiovascular, antioxidant, antiaging effects as a medicinal herbal remedy, however it could also enhance the cell-mediated immune responses [23–28]. Furthermore, *U*. *dioica* is used in the anemia, hypoglycemia, and arthritis treatment, also along with viral infections [29–31].

Different researches in this field have demonstrated that one of the major strategies established by the *Leishmania* parasite is T-helper 1 cytokines inhibiting like IFN-γ, and inversely inducing IL-4 by parasite infected host cells. This immune system cytokines’ imbalance reflects an underlying alteration from T-helper 1 to T-helper 2 responsiveness characterizing susceptibility to *Leishmania* parasite in mice models [32, 33]. On the other hand, susceptibility/resistance to *Leishmania* parasite infection in BALB/c mice directly associates with the main dominance of IL-4 driven TH2 responses terminating infection or an IFN-γ dominated TH1 response that would result in healing along with parasite clearance. However, host macrophage cells can intensely control *Leishmania* infection under suitable conditions, where the major effector mechanism required is initiated by the Th1-type cytokines releasing, especially IFN-γ and tumor necrosis factor. These effectors considerably stimulate the release of NO from host macrophages throughout inducible NO synthase (iNOS), which is the main pathway responsible for clearance/killing parasites [32–34].

This is the first detailed study accomplished on investigating the antiparasitic characteristics of *U*. *dioica* aqueous extract against CL due to *L*. *major*, both *in-vitro* and in the BALB/c mouse model of cutaneous leishmaniasis, including measuring the murine host immune responses.

## Materials and methods

### Materials

Dimethylsulfoxide (DMSO), L-glutamine, adenosine, gentamicin, adenosine, HEPES, hemin and Amphotericin B, were bought from Gibco (Gibco, Life Technologies GmbH, Karlsruhe, Germany) and Sigma (Darmstadt, Germany). 3-(4,5 dimethylthiazol-2-yl)-2,5-diphenyl tetrazolium bromide (MTT) was obtained from Sigma-Aldrich (Deisenhofen, Germany). Schneider’s Drosophila medium, M199 medium, Dulbecco’s modified Eagle’s medium (DMEM) phenol red-free, RPMI-1640, Fetal Calf Sera (FCS) were supplied from Gibco (Gibco, Invitrogen Corporation, Carlsbad, CA).

### Preparation of plant extract

**Fig 1:**
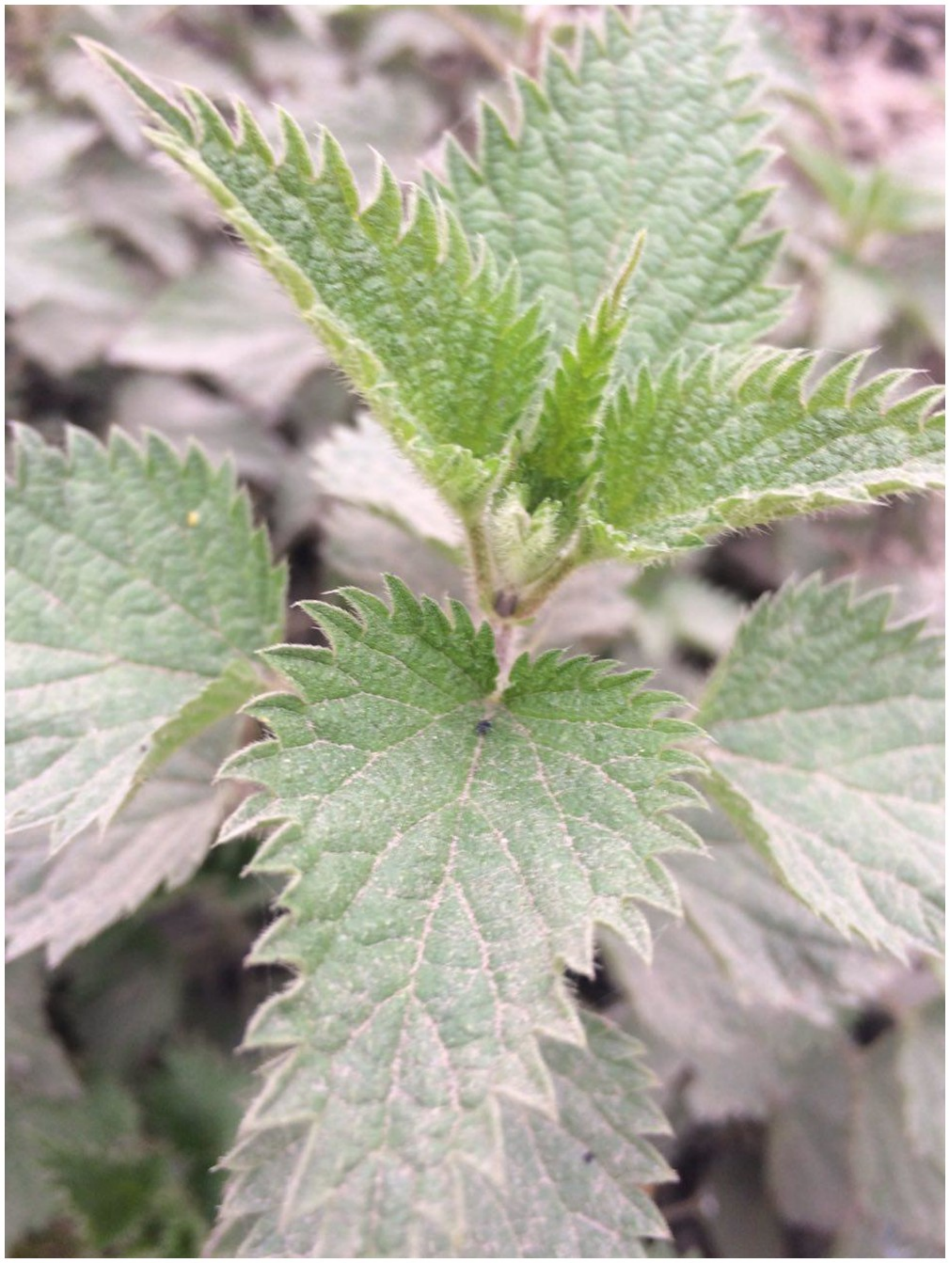
*Urtica dioica* L. (stinging nettle or Urticaceae) isolated from Amol city (Mazandaran Province), which is located on the Haraz River banks during the summer 2017.

The *U*. *dioica* leaves as shown in Fig 1 were obtained during the summer 2017, from the mountainous region of Amol city (Mazandaran Province, northern Iran), which is located on the Haraz River banks (26 25'N 52 21'E) at an altitude of 76 m above the sea level. The plant species were identified by a botanist in Shahid Beheshti Medical Herbarium Center and Agricultural Research Center. The specimen voucher number was 2365. Extraction process was performed with respect to the method accomplished by Domínguezet A. *et al*. [35]. After that, the aqueous extraction processes of powdered materials of *U*. *dioica* were applied, with respect to the method that was described earlier [36]. The material was sterilized throughout filtration by a membrane filter (0.22 μm). The extract was utilized fresh, and was also prepared at different concentrations in order to evaluate its antileishmanial activity.

### Macrophage and parasite cultures

The mouse macrophage cell line J774A.1 (ATCC TIB-67^™^) was purchased from Pasteur Institute of Iran (Tehran, Iran), and was applied in this study. The cell line was cultured in RPMI-1640 supplemented with 10% fetal calf serum, which contained 0.1 mg/mL of streptomycin and 100 IU/mL penicillin G. J774A.1 cells kept at 37°C in 5% CO2 (pH 7.6). The passage of the J774A.1 cell line was accomplished every three days. *L*. *major* (MRHO/IR/75/ER) was purchased from Pasteur Institute of Iran (Tehran, Iran) and cultured at 25°C in RPMI-1640 with 10% fetal calf serum (pH 7.4). The *Leishmania* parasite was stored in a highly virulent state throughout a continuous passage in mice (BALB/c).

### *In-vitro* cytotoxicity assay of the extract on macrophages (CC_50_)

In order to determine the cytotoxic effects of *U*. *dioica* extract, the J774A.1 cells (5×10^4^ cells) were seeded in the presence of the extract growing concentrations (10 to 100000 μg/ml) in 96-well microliter culture plates (Orange Scientific, E.U.) in 5% CO2 for 48 h at 37°C. Cell viabilities were measured using colorimetric methyl thiazole tetrazolium (MTT) assay as it was described earlier [37]. Absorbance ratios were quantified at O.D. 570-nm. PBS and amphotericin B were utilized as controls. These results were indicated as the mean percentage (%) reduction of macrophages in comparison with untreated control samples × 100. Finally, the concentration producing 50% cytotoxicity (μg/ml) (CC_50_) was determined with respect to the method performed by Weislow *et al*. [38]. CC50 values were calculated using Prism 5.0 software (Graph-Pad Prism, San Diego, California, USA).

### *In-vitro* cytotoxicity assay of the extract on promastigotes (IC_50_)

The effects of rising concentrations of *U*. *dioica* extract (10 to 100000 μg/ml) on the stationary phase of *L*. *major* promastigotes (2.5×10^6^ parasites) were quantified for 48 h at 26°C. The susceptibility was determined by the use of colorimetric yellow MTT assay [37] and the extract concentration was estimated, which was resulted in 50% inhibition in promastigotes growth (μg/ml) (IC50).

### *In-vitro* cytotoxicity assay of the extract on infected macrophages by *Leishmania* (EC_50_)

J774A.1 mice macrophages (5×10^4^ cells) were cultured onto a crystal chamber slide (Thermo Scientific Pierce Chemical Co, Massachusetts, USA), along with in 9a 6-well culture plate in RPMI-1640 medium supplemented with 10% fetal calf serum, which was incubated for 24 h at 37°C in 5% CO2. *L*. *major* promastigotes were cultured at 25°C in RPMI-1640 with 10% fetal calf serum (pH 7.4); also after reaching the stationary growth phase, they were used to infect the J774A.1 macrophages, throughout adding to each chamber slide and 96-wells with parasite-to-host cell ratio of 1:10 followed by incubation for 24 hours. Soon after that, the free promastigotes were removed by washing three times using serum-free RPMI-1640 medium. Lastly, infected macrophages were treated with increasing concentrations (10 to 100000 μg/ml) of *U*. *dioica* extract for 48 h at 37°C in 5% CO2 as it was described earlier [39, 40].

### Selectivity Index (SI) Determination

At this stage, the ratio of the reported CC50 value of the cytotoxic activity to the reported EC50 value of the antileishmanial activity was determined in order to calculate the extract selectivity index (SI) [41]. Furthermore, SI was calculated for promastigote forms of parasite (SI = CC_50_ Macrophages/IC_50_ promastigote) [42]. At a time that the SI value is under 10, that compound indicates ideal antileishmanial activity. On the other hand, the ideal herbal compounds would be cytotoxic solely at very high concentrations, and have antileishmanial activity at very low concentrations (higher reported values = greater extract activity) [41].

### Parasite Rescue and Transformation Assay (PRTA or transformed promastigotes)

The infected J774A.1 macrophages were washed for two times with fetal-calf-serum-free RPMI-1640 medium in order to remove the medium remnants. A total of 25 μl of RPMI-1640 medium containing SDS (0.05%) was added to each well for the infected macrophages lysis. The plate was shaken for 30s, and Schneider’s Drosophila medium supplemented with 10% fetal calf serum was added to each one of the wells. The plate was incubated for 72 h at room temperature (26°C), in order to transform the rescued amastigote forms to promastigotes. The antileishmanial effects of the *U*. *dioica* extract was assessed by applying MTT techniques after measuring the effective concentration (EC50: 50%, μg/ml) [43].

### Ethical statements

All animal experimental procedures of this study were approved by the Human and Animal Research Ethics Committee of Shahid Beheshti University of Medical Sciences (ethical code: IR.SBMU.MSP.REC.1397.515). This study was accomplished with respect to the guidelines of the Specific National Ethics for Biochemical Research issued by the Research and Technology Deputy of the Ministry of Health and Medical Education (MOHME) of Iran (issued 2005).

### Mice

All attempts were made in order to reduce mice suffering throughout the experiment course. BALB/c mice (Female; 4–6 weeks old; weigh 20 g) were bought from Pasteur Institute of Iran. All the mice were kept in ventilated-plastic cages and housed in a controlled animal care facility including 23±2 °C, humidity 55–60%, and 12 h of light-dark cycles with free access to an adequate amount of food and tap water.

### Safety study of *U*. *dioica* extract on mice model

To test the *U*. *dioica* extract effects on BALB/c mice, the toxicity of the extract was determined. The animals were divided into seven separate groups: G_A_, G_B_, G_C_, G_D_, G_E_, G_F_, and G_G_ (four mice per each group) (Table 1). As indicated in Table 1, group G_A_ was control group (received no treatment); groups G_B_, G_C_ and G_D_ received *U*. *dioica* intramuscularly (IM) with three increasing concentrations of 150 mg/kg, 200 mg/kg, and 250 mg/kg; and GE, G_F_, and G_G_ received the extract intralesionally (IL) with three rising concentrations of 150 mg/kg, 200 mg/kg, and 250 mg/kg, respectively. All mice were monitored for about one week and their vital signs like sound sensitivity, body weight, diarrhea, sleepiness, and shedding of hair were carefully observed after the herbal therapy [44].

**Table 1:** Standard safety assessment of *U*. *dioica* L. aqueous extract in BALB/c mice with two different injection routes, intramuscular (IM) and intralesional (IL).

Groups (G)	Treated with	Concentrations (mg/kg)	Route of administration
**GA**	No treatment	-	-
**GB**	*U*. *dioica*	150	IM
**GC**	*U*. *dioica*	200	IM
**GD**	*U*. *dioica*	250	IM
**GE**	*U*. *dioica*	150	IL
**GF**	*U*. *dioica*	200	IL
**GG**	*U*. *dioica*	250	IL

### Parasite strain and mice infection

The Iranian promastigotes of pathogenic strain of *L*. *major* (MRHO/IR/75/ER) were applied in this study. The *Leishmania* parasite was retained in a strong virulent state throughout a continuous passage in susceptible BALB/c mice. The swollen lymph node tissues from infected susceptible BALB/c mice were isolated and cultured at 26°C in RPMI 1640 medium (pH = 7.2) supplemented with 5% fetal calf serum containing gentamicin (50 μg/ml). The *Leishmania* promastigotes were sub-cultured and monitored for two times per day. For mice infection with *Leishmania* parasite, promastigotes (2.5×10^6^) from stationary phase were isolated and after that inoculated in the left hind footpad subcutaneously (SC). For *L*. *major* antigens preparation, frozen/thawed procedure was used, which was described earlier [44].

### Schedule of mice infection by *L*. *major* and treatment with *U*. *dioica* extract

As indicated in Table 2, for mice infection with *L*. *major* parasite, 9 groups (n = 10) were selected as followings: G_1_: infected, no treatment control; G_2_: infected, positive control (treated with amphotericin B: 8 mg/kg injected by intraperitoneal (IP) route); G_3_: infected (treated with PBS 1X injected by IM route); G_4_, G_5_, and G_6_ infected groups (treated with elevating concentrations of *U*. *dioica* 150 mg/kg, 2000 mg/kg and 250 mg/kg, injected by IM route), G_7_, G_8_ and G_9_ infected groups (treated with *U*. *dioica* ascending concentrations 150 mg/kg, 200 mg/kg and 250 mg/kg, injected by IL route, respectively). By passing thirty days from infection by *L*. *major*, G_2_ group received amphotericin B (IP route) per each day (two times a day for the duration of 14 days), G_4_, the G_5_, G_6_, G_7_, G_8_, and G_9_ groups received increasing U. *dioica* concentrations (150, 200, and 250 mg/kg,) by different routes of IM and IL, respectively (three times per week for 30 days).

**Table 2:** Different BALB/c mice groups for *in-vivo* study evaluation; The treatment, rising concentrations, and two different injection routes including intramuscular (IM) and intralesional (IL) of *U*. *dioica* L. aqueous extract have been separately elucidated for each group.

Groups (G)	Treated with	Concentrations (mg/kg)	Route of administration
**G1**	No treatment	-	-
**G2**	Amphotericin B	8	Intraperitoneal (IP)
**G3**	PBS	1X	IM
**G4**	*U*. *dioica*	150	IM
**G5**	*U*. *dioica*	200	IM
**G6**	*U*. *dioica*	250	IL
**G7**	*U*. *dioica*	150	IL
**G8**	*U*. *dioica*	200	IL
**G9**	*U*. *dioica*	250	IL

Moreover, footpad lesion size (parasite injection site) was monitored each week through parasite infection and/or treatment courses, and was also recorded up to the end of the experiment (about eight weeks). The increased footpad thickness and width for all groups were evaluated using metric caliper.

Immediately after the period of treatment by amphotericin B (14 days) and treatment by *U*. *dioica* extract (30 days), 5 mice from each group (all control and treated groups) were scarified by cervical dislocation, where the spleens, footpads, and lymph nodes were isolated and evaluated for parasite load, arginase enzymatic activity, and cytokine levels (nitric oxide (NO), IL-4 and IFN-γ). The experiment was repeated one month after the treatment by *U*. *dioica* for former groups.

### Quantification of *Leishmania* parasite load using real-time PCR and limiting dilution assay

By passing eight weeks from infection, the *Leishmania* parasite load in the draining lymph nodes was quantified by real-time PCR. Also, the whole parasite DNA was extracted via genomic DNA Extraction Kit (Qiagen, Germany) with respect to manufacturer’s protocols after homogenization of drained lymph nodes. Extracted DNA purity and concentration was determined by the use of NanoDrop spectrophotometer (ND-1000, USA). Accordingly, about 30 ng of extracted genomic DNA was used for this experiment. Absolute real-time PCR was performed using two sets of primers which targeted a specific region of *Leishmania* DNA (kinetoplastid minicircle DNA) including RV1 and RV2 primers (F: 5′-CTTTTCTGGTCCCGCGGGTAGG-3′ and R: 5′-CCACCTGGCCTATTTTACACCA-3′). *L*. *major* total genomic DNA was serially diluted in eight-fold dilutions corresponding to 2×10^7^ parasites in order to generate the standard curve, as it was described earlier [44, 45]. Also it is noteworthy to state that all PCR reactions were performed in duplicate.

Furthermore, five mice from each group were killed. Next, from drained lymph nodes in the culture media, non-motile amastigotes were transformed to the motile active promastigotes and finally were counted microscopically. Parasite load was assessed throughout limiting dilution assay test as it was described earlier [45].

### Cytokine assay

Inductions of two important immune system cytokines, IL-4 and IFN-γ, were quantified in the supernatant of mouse splenocytes [44]. By passing Eight weeks from infection (after end of the treatment), five treated mice from each group were killed where their spleens were homogenized in DMEM phenol red-free medium supplemented with 5% inactivated fetal calf serum. The splenocytes suspension was treated with ACK lysis buffer (Na2EDTA 0.1 mM; NH4CL 0.15 M; KHCO3 1mM) for 5 minutes at 25ᵒC for the purpose of removing the red blood cells. The cells were washed for three times in DMEM phenol red-free throughout centrifugation at 580 g for 8 minutes. Also, splenocytes were enumerated using Trypan Blue in order to quantify the cell viability. The cells were cultured at a density of 3×10^6^ cells/well in a flat-bottomed plate in the medium presence alone, concanavalin A, or *L*. *major* frozen/thawed antigen. The plates were incubated at 37°C in 5% CO_2_ for 5 days. The production of IL-4 and IFN-γ in the supernatants was quantified by the use of ELISA kits (R&D, Minneapolis, MN, USA), with respect to the manufacturers protocol.

### Arginase activity and nitric oxide (NO) measurements

Four weeks post treatment, the enzymatic activity of arginase was quantified in the killed mice groups’ footpads. This test assessed the L-arginine conversion to L-ornithine by applying the microplate method, as described elsewhere [46–48]. A total of 100 μl stimulated splenocytes supernatant was mixed with 100 μl of Griess Reagent Kit [0.1 N (1-naphthyl) ethylenediamine dihydrochloride, 1% sulfanilamide in 5% H3PO4], and after that incubated for 10 minutes at room temperature. In addition, the colored complex absorbance (azo dye) was assessed at OD = 570 nm. The NO absorbance values of each sample were assessed in terms of nitrate standard curve.

### Statistical analysis

Statistical analyses of all cytotoxicity assays including CC50, EC50, and IC50 were performed by the use of Prism 5.0 software (Graph-Pad Prism, San Diego, California, USA). The statistics were analyzed throughout both one-way ANOVA and Student’s t-test. The association between the IFN-c/IL-10 induction and differences in parasite load were calculated using Spearman correlation method. Furthermore, Statistical differences were assumed significant at p-values less than 0.05. Also it is noteworthy to state that all reactions of this study were performed in triplicate.

## Results

### Cytotoxicity effects of *Urtica dioica* on macrophages (CC_50_)

**Fig 2:**
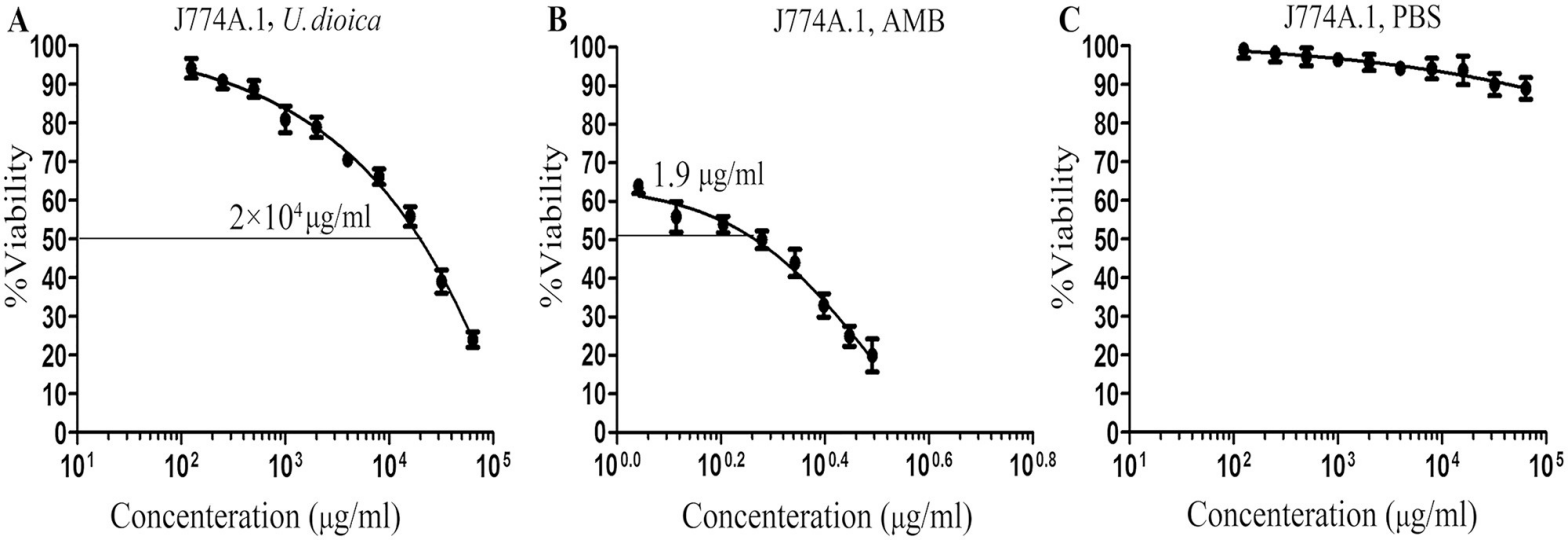
Cytotoxicity assay (CC_50_) of increasing concentrations of *Urtica dioica* L. aqueous extract on mouse J774A.1 macrophage cell line after 48 hours using MTT method *in-vitro*; (A) *U*. *dioica* extract has been toxic only for J774A.1 macrophages at high extract concentrations (CC_50_: 20,000 μg/ml). (B) amphotericin B (CC_50_: 1.9 μg/ml) and (C) PBS were applied as positive and negative controls, respectively. Accordingly, all data have been reported as the mean ± SD of triple repeated experiments. CC50 μg/ml values were calculated from the dose response curve using Prism 5 software.

To test the *U*. *dioica* extract (Fig 1) effects on J774A.1 macrophages cell line, cytotoxicity concentration or CC_50_ (μg/ml) values were determined using MTT method. As shown in Fig 2, *U*. *dioica* extract has been only toxic for J774A.1 macrophages at high extract concentrations. The CC_50_ (μg/ml) values of *U*. *dioica* aqueous extract for J774A.1 cell line and amphotericin B were 20,000 μg/ml and 1.9 μg/ml, respectively (Fig 2).

### Antileishmanial effects of *U*. *dioica* on *Leishmania* promastigotes (IC_50_)

**Fig 3:**
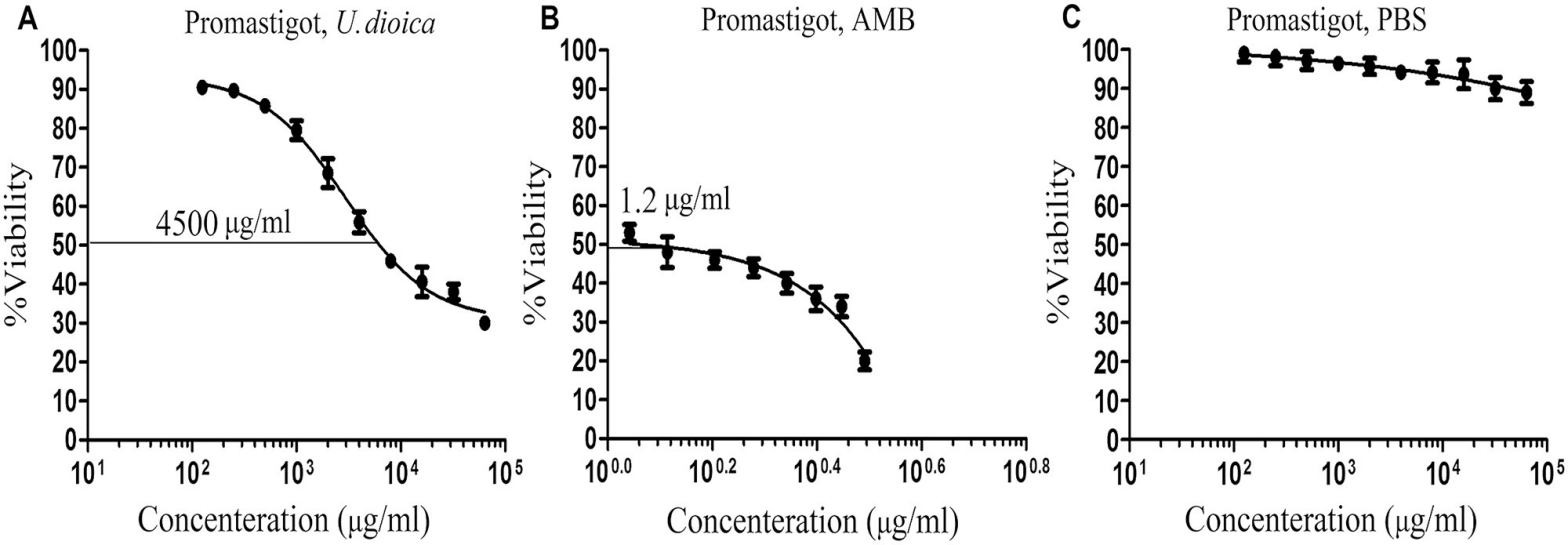
The inhibitory assay (IC_50_) of increasing concentrations of *U*. *dioica* L. aqueous extract on *L*. *major* promastigotes after 48 hours using MTT method *in-vitro*; (A) *Urtica dioica* extract, (B) amphotericin B (AMB), and (C) PBS. All data have been reported as the mean ± SD of triplicate results.

To test the *U*. *dioica* extract effects on *L*. *major* promastigotes, IC_50_ (μg/ml) values were determined using MTT method (Fig 3). The *U*. *dioica* extract strongly inhibited the *Leishmania* parasite growth with the IC50 of 4500 μg/ml (Fig 3A). Amphotericin B, which was evaluated at the concentrations of 1.1 to 3.1 μg/ml as a positive control, could completely inhibit the parasite growth at 1.2 μg/ml (Fig 3B).

### Parasite Rescue and Transformation Assay (PRTA or transformed promastigotes) (EC_50_)

**Fig 4:**
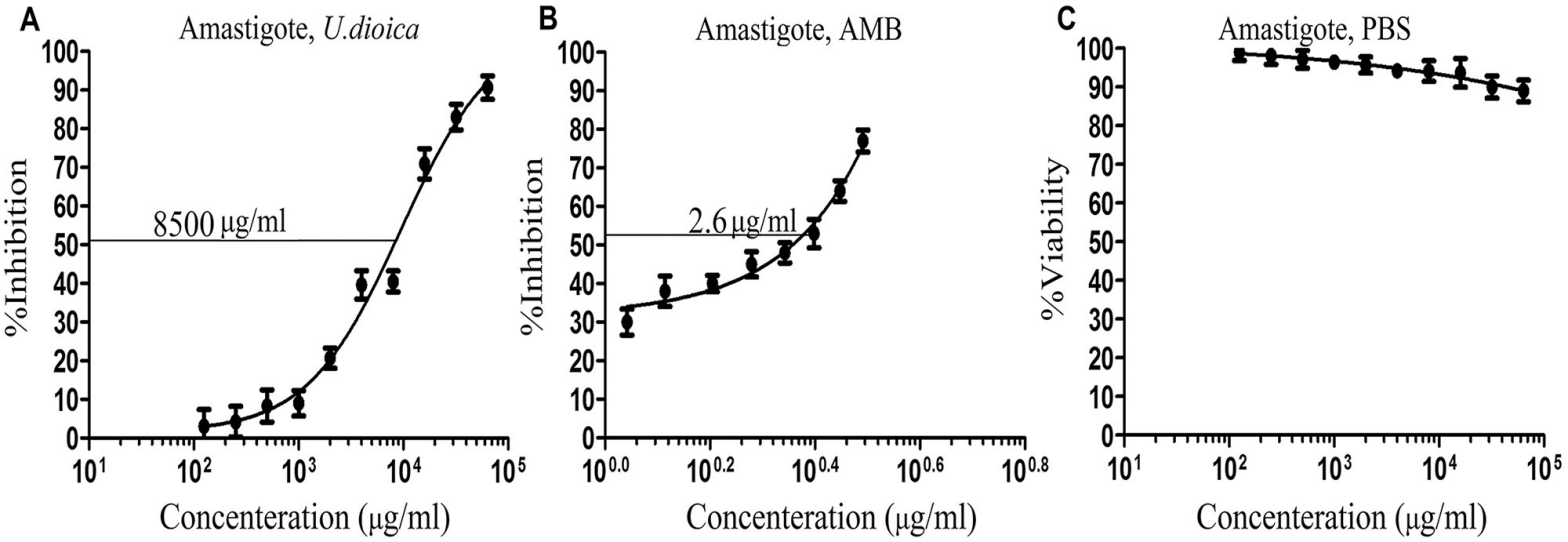
Parasite Rescue and Transformation Assay (PRTA or transformed promastigote) and effective concentration (EC_50_) values of growing concentrations of *Urtica dioica* L. aqueous extract on the transformed promastigote form of *L*. *major* using MTT method *in-vitro*; (A) EC*50* of *U*. *dioica* L. extract against amastigotes form of *L*. *major*, (B) amphotericin B (AMB), and (C) PBS; in accordance with that, all data have been reported as the mean ± SD of triplicate results.

In order to examine the *U*. *dioica* extract effects on *L*. *major* amastigotes growing inside J774A.1 cell line, the EC50 (μg/ml) value was determined by applying the MTT method (Fig 4). As indicated in Fig 4, the *U*. *dioica* extract could inhibit the growth of *Leishmania* amastigotes (EC_50_ 8500 μg/ml) (Fig 4A). For amphotericin B, the EC_50_ was 2.6 μg/ml (Fig 4B). Therefore, *U*. *dioica* extract did not indicate any negative/toxic effects to mice J774A.1 macrophage cell line, but it could inhibit the intracellular amastigotes growth and killed the parasite (Figs 2 and 3).

### *U*. *dioica* extract and selectivity index (SI)

*U*. *dioica* extract was active against the amastigotes of *L*. *major* with a favorable SI (SI = 2.4 μg/ml). Furthermore, the cytotoxicity determination demonstrated that *U*. *dioica* extract was strongly selective against *L*. *major* promastigotes, compared to mammalian J774A.1 cell line with an SI of 4.4 μg/mL. Therefore, the result propose that the extract was highly active against both forms of *L*. *major* promastigotes and amastigotes (SI = 2.4 μg/mL and SI = 4.4 μg/mL, respectively) in comparison with macrophages.

### *U*. *dioica* extract has no toxicity in BALB/c mice

**Fig 5:**
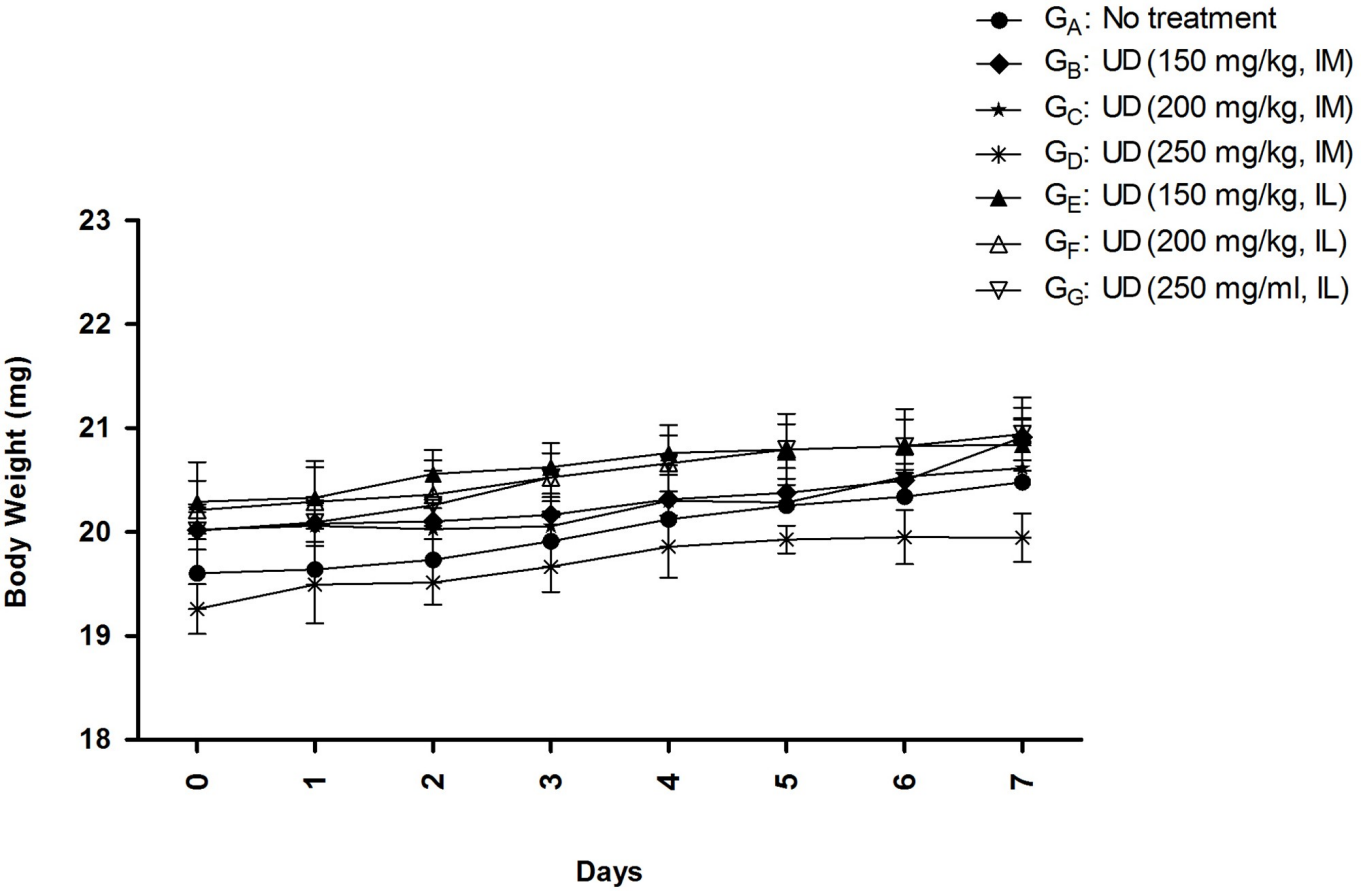
Toxicity assessment of *Urtica dioica* L. (UD) extract in BALB/c mice by measuring the body weight; in regard with that, all data have been reported as the mean ± SD, 3 mice per group.

*U*. *dioica* extract toxicity assessment in BALB/c mice provoked no sign of mortality or toxicity, as measured by changes in the body weight, by passing one-week from extract injection using IM and IL routes as shown in Table 1 and Fig 5.

### Lesion treatment with *Urtica* dioica in infected BALB/c mice

**Fig 6:**
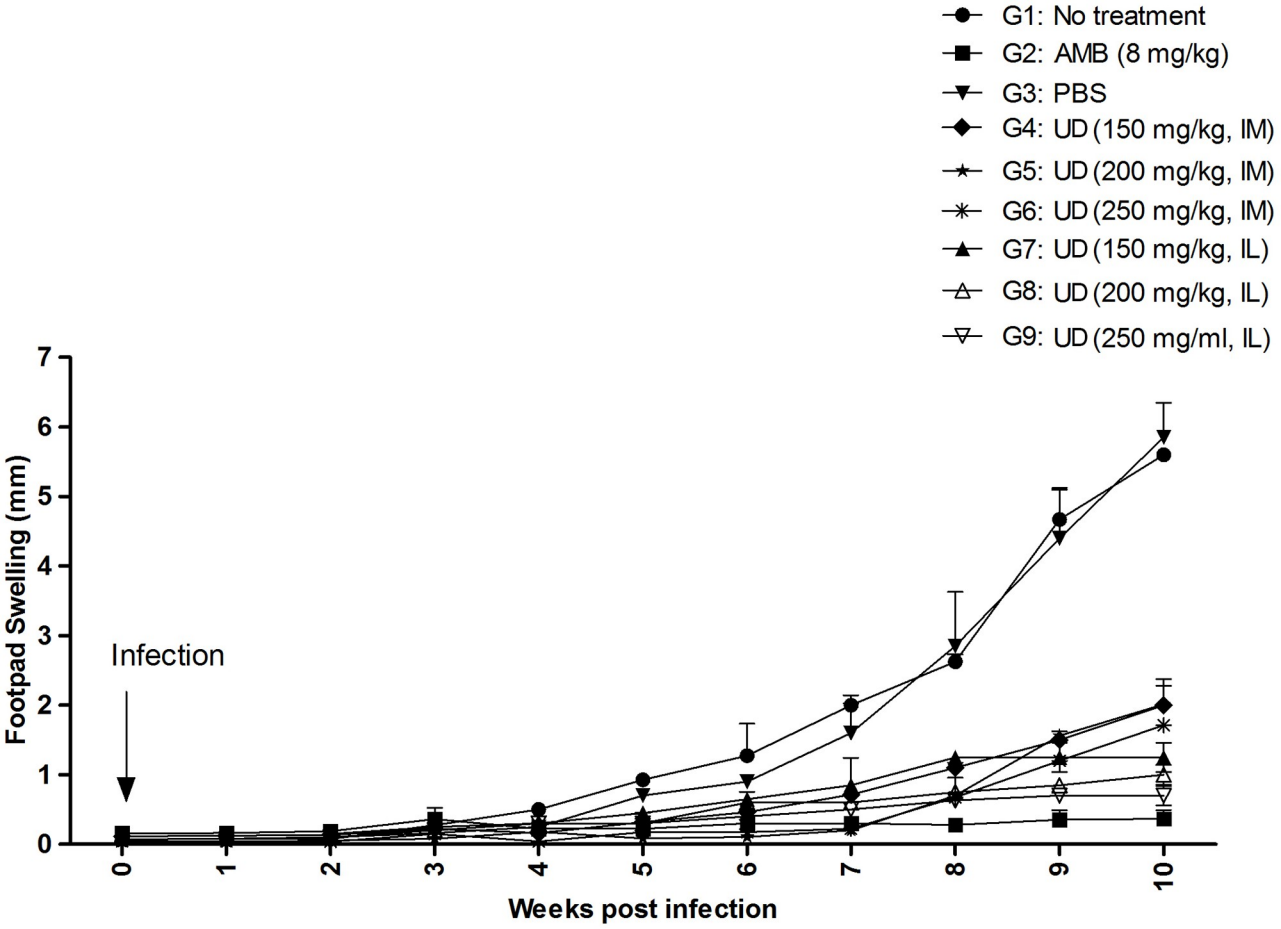
Experimental CL lesion development in BALB/c mice infected SC with promastigotes of *L*. *major* parasites treated with *Urtica dioica* (UD) aqueous extract.

To measure the effect of *U*. *dioica* extract on the experimental CL development, BALB/c mice were infected SC with stationary phase promastigotes of *L*. *major* parasites where CL lesion development was monitored for the duration of 10 weeks. By passing four weeks from treatment with *U*. *dioica* extract groups, all of the IM and IL treatment groups demonstrated remarkably smaller CL lesions in comparison with no treatment and PBS control groups (Table 2 and Fig 6). It is also noteworthy to state that the G_9_, *U*. *dioica* 250 mg/kg IL treatment group, had the smallest CL lesions in comparison with the Amphotericin B treatment group.

### Reduction of parasite load in the BALB/c after treatment with *Urtica dioica* extract

**Fig 7:**
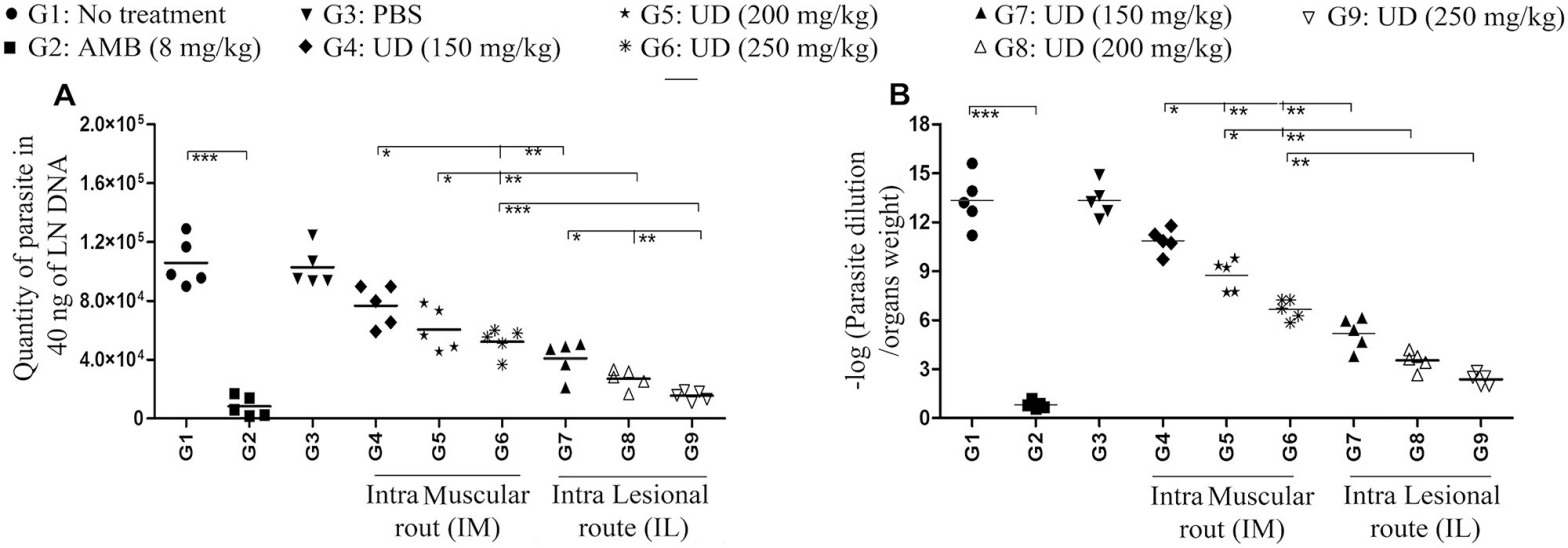
*Leishmania* parasite loads in the draining lymph nodes of BALB/c mice treated with *Urtica dioica* (UD) extract at the time of eight weeks post infection; (A) Micro-titration test / limiting dilution assay and (B) quantitative real-time PCR; accordingly, all data have been reported as the mean ± SD, 5 mice per group. These indicated data are representative of three independent experiments presenting similar outcomes (*P < 0.05, **P < 0.01, ***P < 0.001).

The *L*. *major* load/burden in the BALB/c draining lymph nodes was assessed using limiting dilution assay or micro-titration test(Fig 7A), and quantitative real-time PCR (qPCR) (Fig 7B) by passing eight weeks from infection. As displayed in Fig 7, the *L*. *major* parasite burden was significantly reduced in all experimental groups compared to control groups G_1_ and G_3_, no treatment group, and PBS group, respectively. IL injected groups G_7_, G_8_, and G_9_ had the lowest *L*. *major* parasite loads in comparison with IM injected groups G_4_, G_5_, and G_6_. In addition, G_9_ (U. dioica 250 mg/kg, IL) had the lowest level of *L*. *major* parasite load/burden. It is also noteworthy to state that there was no significant difference between G_9_ (*U*. *dioica* 250 mg/kg, IL) and G_2_ (amphotericin B 8 mg/kg).

### *Urtica dioica* extract affects the immune system cytokine productions in BALB/c mice

**Fig 8:**
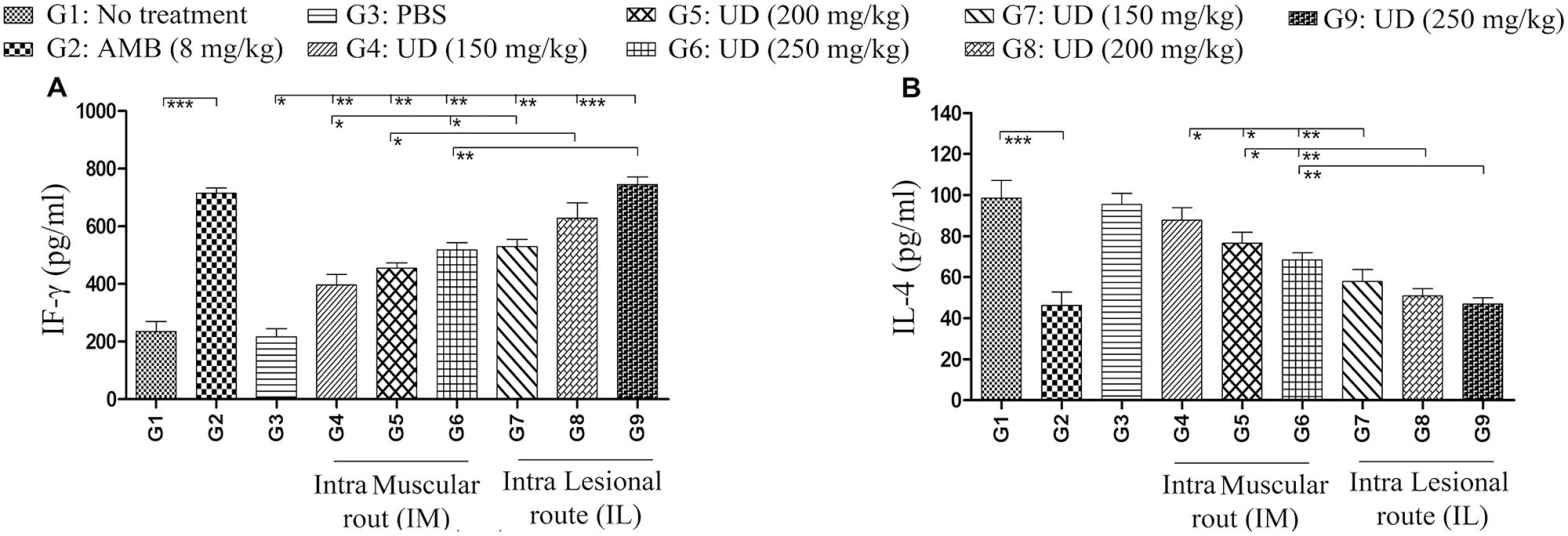
Evaluation of IFN-γ and IL-4 production in the supernatant of frozen/thawed antigen stimulated splenocytes of BALB/c mice in *Urtica dioica* aqueous extract treated groups; (A) IFN-γ (for T helper 1) and (B) IL-4 (for T helper 2) were assessed. In accordance with that, data have been reported as the mean ± SD of 3 independent experiments (*P < 0.05, **P < 0.01, ***P < 0.001).

IFN-γ and IL-4 production in the supernatant of frozen/thawed antigen stimulated splenocytes was assessed by passing eight weeks from infection (Fig 8). Also, Cells stimulated with Concanavalin A were utilized as a positive control. A higher level of IFN-γ was detected in ConA stimulated and in all *U*. *dioica* extract treated groups, which was dose-dependent (Fig 8A). On the contrary, increasing the *U*. *dioica* extract doses elicited lower levels of IL-4 production. (Fig 8B).

**Fig 9:**
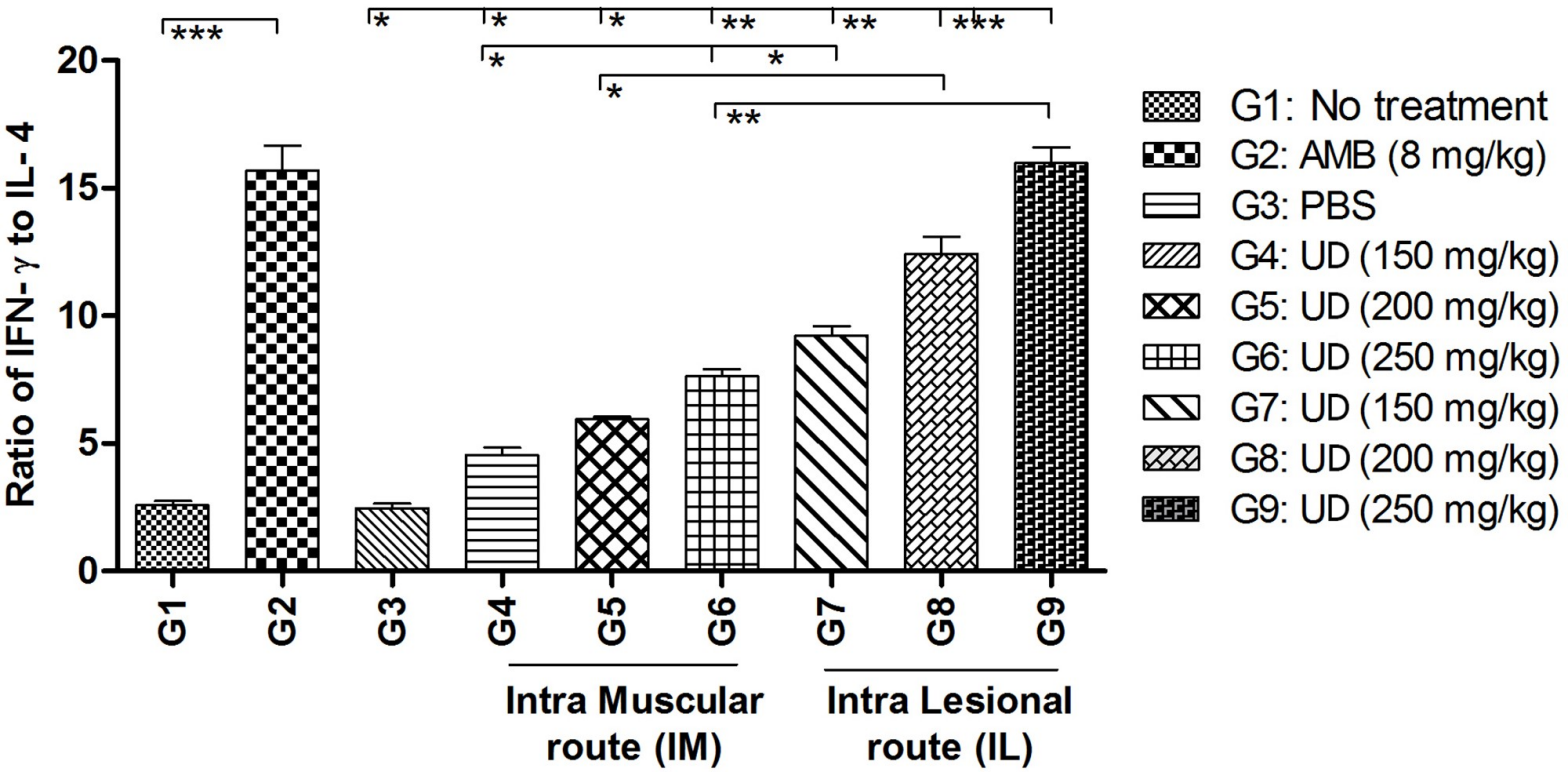
Evaluation of cytokines ratios (IFN-γ/IL-4) in the supernatant of frozen/thawed antigen stimulated BALB/c mice splenocytes in treated groups with *Urtica dioica* extract; Data have been reported as the mean ± SD of 3 independent experiments (*P < 0.05, **P < 0.01, ***P < 0.001).

We also measured the ratio of both cytokines (IFN-γ/IL-4) in all groups (Fig 9). The IFN-γ/IL-4 ratio increased in a dose dependent manner; by considering the route of injection, IL was superior to IM at all of the tested concentrations.

### Arginase activity and NO production in BALB/c treated groups with *Urtica dioica* extract

**Fig 10:**
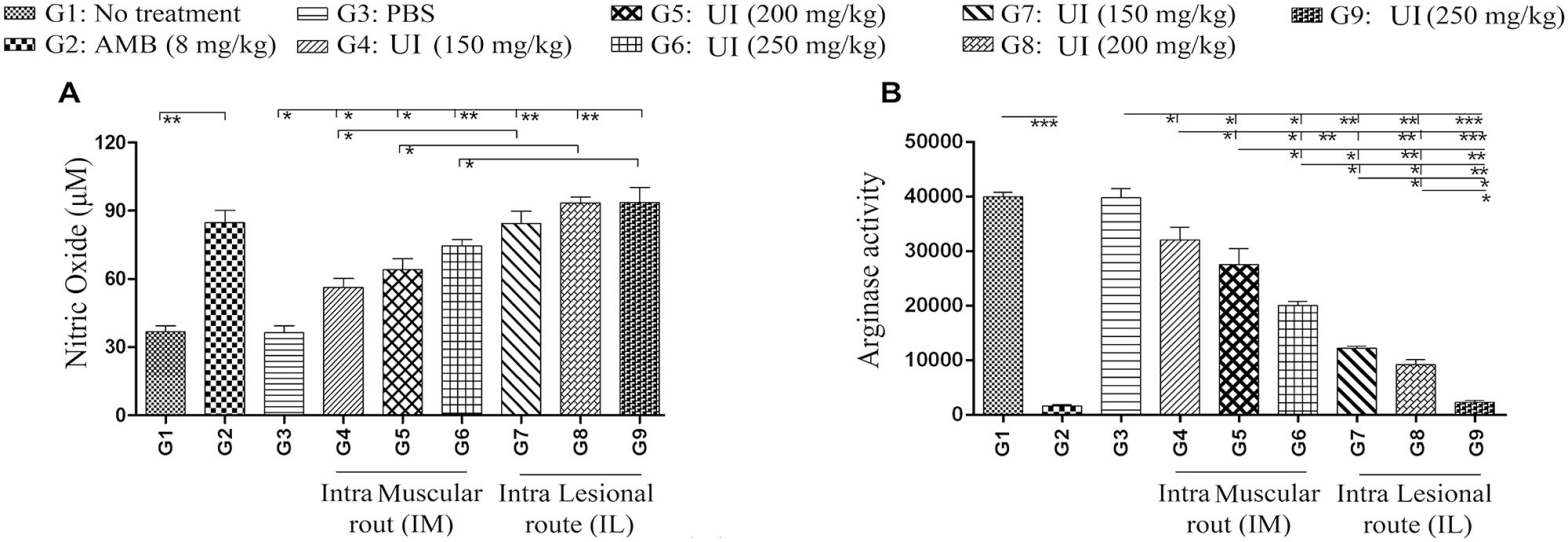
Levels of nitric oxide and arginase in the infected BALB/c by *L*. *major* treated with different *Urtica dioica* extract concentrations; (A) Arginase level (mU/ml) quantified by microplate method; (B) NO production (μM) measured using Griess assay; Data have been reported as the mean ± SD of three independent experiments (*P < 0.05, **P < 0.01, ***P < 0.001).

Arginine is metabolized via two main pathways involving either inducible nitric oxide synthase (iNOS) or arginase inside the host macrophages. Th1 cytokines (IFN-γ) triggers NO production by up regulating iNOS while the Th 2 cytokines (IL-4) increase the arginase activity induction [49]. Killing the intracellular parasites like *Leishmania* inside cells and/or its long-term survival is directly mediated by iNOS and arginase activities, respectively (Fig 10).

All of the experimental groups significantly augmented the NO production, in a dose dependent manner; concerning the route of injection, again IL was superior to IM at all of the tested concentrations (Fig 10A). Arginase activity indicated a clear inversely association with NO levels across all treatment groups (G_4_, G_5_, G_6_, G_7_, G_8_, G_9_, and G_2_, respectively) (Fig 10B). Therefore, this study results provide strong evidence that Th1 cells were activated throughout *U*. *dioica* treatment.

## Discussion

Herbal plants are alternatives in the development of new anti-leishmanial agents. These materials that are found in nature, have been indicated to exhibit a selective action against parasites without host cell viability reducing. They are also more available for isolated populations in comparison with traditional pharmacotherapy [50–54]. Studies propose that there are several natural extracts that have biological functions for clinical applications. Accordingly, one of them can be used as a novel natural compound is *Urtica dioica*. It is identified as a member of the Urticaceae family and is native to Europe and Asia. Although, several randomized, double-blind, placebo-controlled clinical trials have investigated the *U*. *dioica* root extracts effect on allergic rhinitis, arthritis, neuralgia, cardiovascular, and prostate disease [55–58], but its anti-leishmanial activity has not been described yet. Consequently, this research is the first comprehensive study conducted on the therapeutic effects of *U*. *dioica* aqueous extract as an anti-parasitic herbal drug with acceptable immunoregulation of immune cells without excessive toxicity to the host cells and direct anti-leishmanial activity in *in-vitro* and in mice models.

The inhibitory (IC50), cytotoxic (CC50) and effective concentrations (EC50) of *U*. *dioica* extract were assessed in order to determine its anti-leishmanial activity. The achieved results demonstrated that the optimal concentrations of the extract for reducing the promastigotes and amastigotes growth were 3500 and 6000 μg/ml, respectively. These doses killed half of both forms of the parasite. These results indicated that the extract has a potent action against *L*. *major* promastigotes and amastigotes via modulation of immune response, while having no harmful side effects for the host macrophages. Although, the action mechanism throughout which *U*. *dioica* kills *Leishmania* parasites is not identified yet, but a study has indicated that *U*. *dioica* can have a modulatory effect on phase I enzyme systems for A4H activity and the urease pathway [59, 60]. Another study proposed that the *U*. *dioica* extract could inhibit several key inflammatory events and after that cause seasonal allergies symptoms [61].

The selectivity index (SI) is a great indicator of an herbal compound’s activity. The SI of an herbal compound is an extensively accepted parameter that is used in order to express a compound’s *in-vitro* efficacy in the parasite multiplication inhibition [42]. The SI data have indicated that the *U*. *dioica* extract was approximately 2.4 and 4.4 times more toxic to *L*. *major* amastigotes and promastigotes, respectively, in comparison with the mammalian cell. Actually, the extract elucidated a significant antileishmanial activity, and has the ability of reducing the survival rate of intracellular parasites at nontoxic concentrations for the host macrophages.

The efficiency of *U*. *dioica* extract was determined for the treatment of BALB/c mice infected with *L*. *major*. After treatment with *U*. *dioica* extract, all of the treatment groups showed remarkably smaller CL lesions in comparison with no treatment and PBS control groups. The *Leishmania* parasite burden was measured by applying conventional limiting dilution assay (micro-titration), and also by quantitative real-time PCR (qPCR). A significant difference was found in the results of both techniques between the parasite burden in the *U*. *dioica* extract and amphotericin B treated groups, in comparison with both control groups (no-treatment and PBS groups).

It was established that the aqueous extract treatment considerably reduced the *L*. *major* replication and effectively killed them. Also, a marked induction of IFN-γ and NO expression as a reliable cell mediated (Th1) immune system was noted in the treatment groups, especially the IL group, in comparison with the control groups. The most efficient mechanism of parasite death involved the production of IFN-γ and tumor necrosis factor alpha (TNF-α) by CD4+ Th1 cells. These stimulate the synthesis of inducible iNOS, generating NO, a potent cytotoxin involved in the *Leishmania* parasites clearance or inhibition. Earlier researches have proposed that eradication of *Leishmania* using activated macrophage cells (M1) could control the superoxide production and directly leads to death by NO production [62]. The stimulation of effector cells that produce the macrophage-activating cytokines is required in the host, in order to control the parasite propagation. Presumably, this function can be increased by the effects of IFN-γ (as a Th1 cytokine) for production of NO [63].

Recent studies have indicated that L-arginine can be catabolized to urea and polyamines throughout ARG activity and/or NO through iNOS induction in activated macrophages. With respect to these studies, Th2 cytokines like IL-4 stimulate ARG activity and polyamine generation, which can support *Leishmania* proliferation. The leishmanicidal action mechanism exhibited by some natural extracts is associated with the ability to induce the microbicidal response activation in macrophages and promotion of ROS and NO production, which both of them lead to amastigote death [51, 64]. These results demonstrated that the lowest ARG activity and IL-4 production (as a Th2 cytokine) occurred in the treatment groups, especially in the IL group. In some infections associated to *Leishmania*, IL-4 induction (as a Th2 cytokine) and increased the ARG activity with L-arginine metabolism in alternative activated macrophage cells (M2) play a significant role in the infection and parasite proliferation establishing [49]. Also, these findings are in agreement with other studies outcomes [65, 66].

*In-vitro* and *in-vivo* testing demonstrated anti-leishmanial activity of *U*. *dioica* extract where the extract effects increased as the concentration was elevated. This dose-dependent activity is inconsistent with other studies results [67]. Additionally, in this study, intralesional (IL) and intramuscular (IM) routes were used in order to evaluate their effects with *U*. *dioica* extract on those mice infected with *L*. *major*. Surprisingly, the results indicated remarkable changes in the parasite loads, NO production, IFN-γ/IL-4 ratio, and ARG activity. Moreover, the IL groups (G7, G8 and G9) had considerably smaller lesions compared to the IM and control groups. One possible explanation for the inadequate responses to IM therapy for *Leishmania* infection in mice is that only low concentrations of the extract and amphotericin B could reach the CL infection site. Consequently, this discovery provides more evidence for other studies results [68, 69].

Different types of studies have been conducted on effective drugs in order to reduce infection by parasites in all over the world. Some research has been accomplished on the use of herbal medicine on *L*. *major* parasites. Methods with different types of drug delivery and nanoparticle therapy were examined in order to determine the effectiveness of herbal substances and disease treatments by pharmaceutical companies. In Iran, different types of herbal medicine have been considered for treating the *L*. *major* infection. Therefore, No documented reports far have investigated the anti-leishmanial effects of *U*. *dioica* extract in a mice model. This extract is a promising herbal drug candidate and a novel alternative treatment for CL caused by *L*. *major* as it induces Th1 responses. As a result, this study offered satisfying results in the parasite eradicating in both *in-vivo* and *in-vitro* experiments. It can be concluded that *U*. *dioica* extract could be an ideal candidate for the isolation and production of novel drugs against this neglected tropical disease and its promising effects for future studies.
